# Is Ovarian Tissue Transplantation Safe in Patients with Central Nervous System Primitive Neuroectodermal Tumors?

**DOI:** 10.3390/jcm9124101

**Published:** 2020-12-18

**Authors:** Thu Yen Thi Nguyen, Alessandra Camboni, Rossella Masciangelo, Jacques Donnez, Marie-Madeleine Dolmans

**Affiliations:** 1 Gynecology Research Unit, Institut de Recherche Expérimentale et Clinique, Université Catholique de Louvain, Av. Mounier 52, 1200 Brussels, Belgium; thu.nguyen@uclouvain.be (T.Y.T.N.); alessandra.camboni@uclouvain.be (A.C.); rossella.masciangelo@uclouvain.be (R.M.); 2 Department of Anatomopathology, Cliniques Universitaires Saint-Luc, Av. Hippocrate 10, 1200 Brussels, Belgium; 3 Society for Research into Fertility, Av. Grandchamp 143, 1150 Brussels, Belgium; jacques.donnez@gmail.com; 4 Department of Gynecology, Cliniques Universitaires Saint-Luc, Av. Hippocrate 10, 1200 Brussels, Belgium

**Keywords:** ovarian tissue cryopreservation, ovarian tissue transplantation, primitive neuroectodermal tumors, minimal disseminated disease, neuron-specific enolase, glial fibrillary acidic protein

## Abstract

The risk of reseeding malignancy harbored in cryopreserved and transplanted ovarian tissue has been a source of concern. This study aimed to determine the potential relationship between frozen–thawed ovarian tissue transplantation and primary cancer recurrence. Three patients with cerebral primitive neuroectodermal tumors (PNET) were included in this study. One woman gave birth to three healthy babies following reimplantation of her cryopreserved ovarian tissue, but subsequently died due to cancer relapse six years after ovarian tissue transplantation. The second subject died from progressive cancer, while the third is still alive and awaiting reimplantation of her ovarian tissue in due course. Frozen ovarian cortex from all three patients was analyzed and xenotransplanted to immunodeficient mice for five months. Main outcomes were the presence of cancer cells in the thawed and xenografted ovarian tissue at histology, immunostaining (expression of neuron-specific enolase and glial fibrillary acidic protein (GFAP)), and reverse-transcription droplet digital polymerase chain reaction (RT-ddPCR) (levels of enolase 2 and GFAP). In conclusion, no malignant cells were detected in ovarian tissue from patients with PNET, even in those who experienced recurrence of the disease, meaning that the risk of reseeding cancer cells with ovarian tissue transplantation in these patients can be considered low.

## 1. Introduction

These days, a cancer diagnosis is no longer a death sentence for most patients. Indeed, anticancer treatments have become increasingly effective, yielding significant improvements in patient survival rates. A more recent focus has become the quality of life of survivors, because these treatment modalities, especially high-dose chemotherapy, pose a threat to a woman’s reproductive organs, leading to premature ovarian insufficiency and subsequent infertility [1,2]. For this reason, an appropriate approach to fertility preservation is required prior to therapy.

For prepubertal patients and women who need to start treatment immediately, ovarian tissue cryopreservation offers a unique option [1,3]. Frozen–thawed ovarian tissue can be transplanted back to the pelvic cavity once cancer therapy is complete and the patient shows no signs of relapse [3]. This procedure results in restoration of ovarian activity in up to 93% of cases, with ovarian function maintained for four to five years [4,5], and sometimes up to seven years [6], depending on the follicle reserve before cryopreservation. By 2018, there had been more than 130 live births reported after autotransplantation of ovarian tissue worldwide [2], and that figure has probably exceeded 200 by now [7].

Safety issues surrounding reimplantation of ovarian tissue from cancer patients have been a cause of concern for many years [8,9]. A number of studies have investigated the risk of reintroducing malignant cells together with the frozen–thawed ovarian tissue, which could induce recurrence of the primary tumor. Malignant cells have been detected in case of leukemia and borderline ovarian tumors, but minimal disseminated disease (MDD) has not been documented in ovarian tissue from patients with bone and soft tissue sarcoma or low-grade breast cancer [10,11,12,13]. According to global transplantation data, no relapses have been recorded in any site associated with ovarian tissue reimplantation [14].

Thirty-one women with neurological malignancies have so far had their ovarian cortex stored in our ovarian tissue biobank, accounting for 5% of indications for ovarian tissue cryopreservation. The present study reports three cases of central nervous system (CNS) primitive neuroectodermal tumors (PNET), including one subject who relapsed six years after ovarian tissue transplantation that resulted in the birth of three children. The second subject unfortunately died. The third is alive and awaiting transplantation of her ovarian tissue in the future. However, her ovarian tissue was collected one month after insertion of a ventriculoperitoneal (VP) shunt, which may increase the risk of cancer cell contamination of the peritoneal cavity [15]. Our aim was to determine the potential relationship between ovarian tissue transplantation and primary cancer recurrence by detecting the presence of malignant cells in cryopreserved ovarian tissue.

## 2. Experimental Section

### 2.1. Patients

Three subjects with PNET were included in the study. The first patient conceived naturally and gave birth to three children as a result of ovarian tissue reimplantation, but she died due to primary cancer relapse, 6 years after the procedure. The second died as her cancer progressed. The third is still alive and disease-free. Use of human tissue from the patients was approved by the Institutional Review Board of the Université Catholique de Louvain on 2 June 2014 (IRB reference 2012/23MAR/125, registration number B403201213872).

### 2.2. Thawing of Frozen Ovarian Strips

The patients’ ovarian cortex was stored in the biobank. Cryovials were thawed according to a previously described protocol [12]. Thawed strips were assigned for MDD testing and xenografting.

### 2.3. Xenografting

Female severe combined immunodeficient (SCID) mice (Charles River Laboratories, France), aged 6 weeks, were used for the present study. Animal welfare was respected by following guidelines approved by the Committee on Animal Research of the Université Catholique de Louvain on 19 June 2014 (reference 2014/UCL/MD/007). Appropriate housing and breeding conditions were strictly applied, as previously reported [16]. The surgical procedure has already been described [11]. The mice were raised in sterile conditions and received frequent health checks. After 5 months’ grafting, euthanasia was performed by cervical dislocation, and grafted ovarian tissues were collected and tested for MDD by histology, immunohistochemistry (IHC), and reverse-transcription droplet digital polymerase chain reaction (RT-ddPCR), as described below.

### 2.4. Histological Analysis

A small fragment of frozen–thawed ovarian tissue was fixed in 4% formaldehyde and embedded in paraffin wax. Samples were serially sectioned every 5 µm and placed on microscopy slides. Every fifth section was stained with hematoxylin and eosin (H&E) (Merck, Darmstadt, Germany), for histological analysis, to determine the presence of metastatic cells in ovarian fragments, as well as follicle viability before and after grafting. H&E slides of primary tumors were used for comparative purposes to detect malignant cells in ovarian tissue.

### 2.5. Immunohistochemical Analysis

Samples from primary tumors were obtained from the anatomopathology department, to serve as positive controls. Negative controls issued from ovarian tissue biopsies from patients with benign uterine pathologies. Expression of the neuronal marker neuron-specific enolase (NSE) and glial fibrillary acidic protein (GFAP) were investigated on patient CNS tumor samples and cryopreserved and post-grafted ovarian tissue. Immunohistochemical staining for NSE and GFAP were automatically performed, using Ventana’s ultraView Universal DAB detection kit on the BenchMark ULTRA IHC/ISH system (Roche, Basel, Switzerland). Rabbit primary NSE antibody (catalog number A598, lot 106, Dako Corporation, CA, USA) and rabbit polyclonal GFAP antibody (catalog number CP040A, B, C, lot 121806, Biocare Medical, CA, USA) were used. Tissue sections were subsequently incubated with ultraView horseradish peroxidase-conjugated multimer antibody reagent (Igs, Ventana Medical Systems, AZ, USA) and counterstained with hematoxylin.

### 2.6. Sample Storage, RNA Extraction and Reverse Transcription

Ovarian strips destined for molecular analysis were cut into small pieces, immediately submerged in 700 µL RNAlater RNA stabilization reagent (Qiagen, Ambion, TX, USA), and stored at −20 °C until use. RNA extraction from ovarian tissue was performed by using the RNeasy Plus mini kit (catalog number 74104, Qiagen, Germany), following the manufacturer’s instructions. Patient CNS tumor samples embedded in paraffin blocks were processed by using the RNeasy FFPE kit (catalog number 73504, Qiagen, Germany) as instructed by the manufacturer.

Extracted RNA was qualified with the NanoDrop 2000 spectrophotometer (Thermo Fisher Scientific, ND-2000, Wilmington, NC, USA), and purity was checked by assessing A_260/280_ ratios over 1.90, followed by immediate storage at −80 °C. Complementary DNA (cDNA) synthesis was carried out by using the Advantage RT-for-PCR kit (Takara, catalog number 639505, Mountain View, CA, USA) and 0.5 µg total RNA, according to the manufacturer’s protocol. The mixture was then placed in the thermal cycler (Applied Biosystems, serial number 096S9030939, Foster City, CA, USA) for 1 h at 42 °C, 5 min at 94 °C, and 3 min at 4 °C. All cDNA was kept frozen at −20 °C.

### 2.7. Droplet Digital PCR

The patients’ cerebral PNET samples were used as positive controls, to detect enolase 2 (ENO2) fusion transcripts with human primer ENO2 Hs00157360_m1 (Thermo Fisher Scientific, LA, USA) and GFAP (Hs00909233_m1), while ovarian tissue biopsies from ten patients with benign gynecological diseases served as negative controls. Two housekeeping genes used in the study were Abelson murine leukemia viral oncogene homolog 1 (ABL1, Hs01104728_m1) and beta 2 microglobulin (B2M, Hs00187842_m1). Sequences of primers and probes are detailed in Supplementary Materials Table S1.

Droplet digital PCR (ddPCR) was performed on a QX200 system (Bio-Rad Laboratories Inc., Hercules, CA, USA). Each 20 µL reaction mixture consisted of ddPCR^TM^ Supermix for probes (no dUTP) (Bio-Rad Laboratories) for ENO2 assays, 10 ng cDNA, and Tris-EDTA buffer, according to a previously reported protocol [17]. Data were analyzed with QuantaSoft^TM^ software (Bio-Rad Laboratories). Target concentrations in each sample were expressed as ENO2 or GFAP copies per microliter.

### 2.8. Defining the Limit of Blank and Limit of Detection

The limit of blank (LOB) and limit of detection (LOD) of ENO2 and GFAP in human ovarian tissue evaluated by ddPCR were defined [18]. The blank sample consisted of the RNA pool from 10 healthy women’s normal ovarian tissue, measured by testing 60 replicates and calculated by using the following formula: LOB = mean_blank_ + 1.645 (SD_blank_) [19]. The LOD was determined by measuring serial dilution tenfold between patient CNS tumors and blank samples, whereLA each stage of dilution involved 8 replicates and was calculated by using the following formula: LOD = LOB + 1.645 (SD_low concentration sample_) [19]. The LOD serves as a threshold to determine quantification of a marker in ovarian tissue from patients with PNET by ddPCR, namely “detected” or “not detected” malignant cells in a given sample.

### 2.9. Next-Generation Sequencing

As no molecular biology markers were found for Patient 1 by RT-ddPCR, next-generation sequencing (NGS) was performed in the hope of finding a specific gene profile for the disease. The primary tumor sample from Patient 1 was tested for genetic mutations in the glioma panel (including ATRX, BRAF, CDKN2A, CDKN2B, CIC, DAXX, EGFR, FOXR2, FUBP1, H3F3A, HIST1H3B, IDH1, IDH2, KEL, KRAS, LZTR1, MET, MSH6, NF1, NOP53, PDGFRA, PIK3CA, PIK3R1, PTEN, QKI, RB1, TP53, TP73, TSC1, and TSC2), using an NGS technique. The detailed methodology for NGS is not described here. The patient’s ovarian tissue was then checked by NGS when positive mutations were found in the primary tumor.

### 2.10. Statistical Analysis

Results of ddPCR were calculated statistically, using Graphpad Prism, version 8.0, for desktop (GraphPad Software Inc., LA, CA, USA). A *p*-value < 0.05 was considered significant. 

## 3. Results

### 3.1. Patient Information

#### 3.1.1. Patient 1

A 17-year-old patient underwent ovarian tissue cryopreservation, followed by transplantation some years later. In 2001, the patient was diagnosed with supratentorial PNET on the right orbitofrontal lobe of the cerebrum and underwent two operations for tumor removal. Prior to commencing adjuvant chemotherapy, she was offered a laparoscopy to collect ovarian tissue from both ovaries. No histological evidence of malignancy was found in the retrieved cortical and medullary tissue. Ovarian tissue cryopreservation was performed by slow-freezing, yielding seven cryotubes containing four strips each. Thereafter, the patient underwent and completed chemotherapy with 6 VIDE (vincristine, ifosfamide, doxorubicin, and etoposide) cycles according to the Euro-Ewing 99 protocol. In 2002, however, extra-neural PNET metastasis to the right lung was diagnosed, and radical resection was required. This was followed by intensive chemotherapy (two cycles of VAI (vincristine, actinomycin D, and ifosfamide) in association with busulfan–melphalan) and stem cell autotransplantation. Radiotherapy was indicated at the tumor site, at a dose of 45 Gy for 25 sessions, plus a booster of 54 Gy given in five doses. Regular follow-up data revealed complete disappearance of this CNS cancer.

After her treatment, the patient developed secondary ovarian failure, with amenorrhea and elevated serum follicle-stimulating hormone (FSH) levels. She was then (2003) prescribed hormone replacement therapy (HRT) and continued her routine gynecological and oncological follow-up. She got married in 2008 and subsequently came to our gynecology outpatient clinic with a desire to conceive. Examinations and investigations were carried out to explore both gynecological and oncological indications. Her hormone profile three months after stopping HRT showed FSH, luteinizing hormone (LH) and estradiol levels of 45.5 mIU/mL, 16.8 mIU/mL, and <10 pmol/L, respectively, which confirmed her menopausal status. The patency of both fallopian tubes was demonstrated by hysterosalpingography. Transvaginal ultrasound revealed a uterus of 40 × 27 × 17 mm in diameter and endometrial thickness of 1.4 mm with a regular echographic pattern. The right and left ovaries measured 17 × 7 mm and 13 × 6 mm, respectively, and no antral follicles were seen. There were no suspicious masses in the abdomen. The patient was therefore prescribed oral contraceptives (containing 150 µg of desogestrel and 20 µg of ethinyl estradiol, one pill per day) for a period of three months prior to ovarian tissue transplantation to reduce the FSH level [3]. From a neuronal perspective, her follow-up profile included brain magnetic resonance imaging (MRI), which showed her to be disease-free. Laparoscopic surgery for transplantation of frozen–thawed ovarian tissue was performed by senior surgeons (JD and MMD) in November 2008, six years after the patient finished her cancer treatment. During surgery, no malignant masses were detected in the peritoneal cavity, while both ovaries were atrophic (Supplementary Materials Figure S1). They were both decorticated, and the anatomopathological results confirmed an absence of follicles. Ovarian cortical strips (3 × 7 × 1 mm in diameter) were then grafted to the right (seven fragments) and left (six fragments) ovaries.

**Figure 1:**
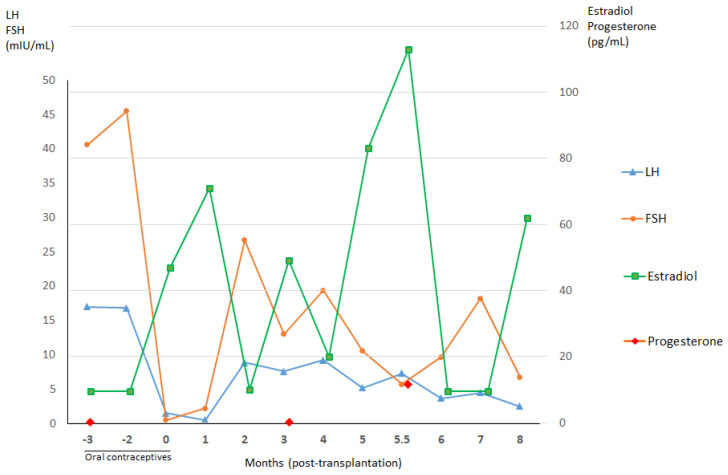
Hormone profiles before and after autografting of frozen–thawed ovarian tissue from Patient 1. The transplantation procedure occurred at zero months. Concentrations of estradiol rose three months after ovarian tissue transplantation with a concomitant drop in follicle-stimulating hormone (FSH), suggesting recovery of gonadal function. These hormone levels reached their peak 5.5 months postoperatively, when progesterone concentrations also increased. The patient subsequently conceived for the first time, nine months post-grafting. LH, luteinizing hormone.

The patient’s hormone profile after ovarian tissue reimplantation is shown in Figure 1. Three and a half months after surgery, a first estradiol peak was detected (49 pg/mL), concomitant with a drop in FSH (13 mIU/mL) and appearance of a follicle at ultrasound. She resumed spontaneous menstrual bleeding. 

Nine months after transplantation, she obtained her first pregnancy by natural conception and gave birth vaginally to a healthy baby boy in April 2010. She went on to have her second and third children in 2011 and 2012, after which she was prescribed oral contraceptives (containing multiphasic combinations of estradiol valerate (1–3 mg) and dienogest (0–3 mg), one pill per day). At the end of 2014, 12 years after her last radio-chemotherapy and six years after ovarian tissue transplantation, the patient relapsed with a nodule on the right frontal lobe of her cerebrum. This nodule was removed in its entirely in January 2015. The anatomopathological result confirmed recurrence of known PNET, without involvement of the cerebral parenchyma. The results of her CNS cancer extension profile, including cerebral MRI, thoracic MRI, positron emission tomography-computed tomography (PET-CT) imaging, and histology of cerebrospinal fluid, were negative. The patient also completed adjuvant radio-chemotherapy involving three cycles of VAI and two cycles of vincristine and ifosfamide during radiotherapy at the tumor site (dose of 54 Gy delivered in 30 fractions of 1.8 Gy for 30 days). In April 2016, she was admitted to hospital by the mobile emergency and resuscitation service, having suffered cardiopulmonary arrest at home after deterioration of her general condition and dyspnea for three days. MRI identified tumor compression of the cervical cord (C2–D1) with leptomeningeal carcinomatosis. After two weeks of treatment, the patient died from medullary compression caused by disseminated PNET. No autopsy was performed at the time, as the family refused.

#### 3.1.2. Patient 2

A three-year-old patient was diagnosed with a PNET on her left frontal-parietal lobe in contact with the meninges, in December 2012. She underwent subtotal resection surgery, but developed multiple meningeal nodular lesions in January 2013. Prior to chemotherapy, her ovarian cortex was retrieved by laparoscopy and cryopreserved, using the slow-freezing technique for fertility preservation. The patient was subjected to seven cycles of chemotherapy, with a protocol for high-grade PNET and autologous stem cell transplantation before complete resection of the tumors in August 2013. She also underwent 17 sessions of radiotherapy in the following month. In October 2013, the subject was admitted to hospital with increased intracranial pressure. MRI revealed new multiple nodules disseminated in supra- and infratentorial regions and enlarged ventricles. An emergency VP shunt was inserted, but the patient died from cardiopulmonary arrest caused by neurological alterations after three weeks of treatment.

#### 3.1.3. Patient 3

The patient was diagnosed with a grade IV PNET in the pineal region at the age of nine and underwent complete resection in August 2008 and insertion of a VP shunt one week later. One month later, laparoscopy was performed for ovarian tissue collection and cryopreservation prior to starting radiotherapy, targeting her tumor bed and cerebrospinal axis with 51 sessions in total. Her hormone profile in October 2018 showed FSH, LH, progesterone, and estradiol levels at 6.6 mIU/mL, 6.0 mIU/mL, 17.8 pg/mL, and 26 pg/mL, respectively. Her first menstrual bleed occurred when she was 17 years of age, and she usually has menstrual periods once a month. She is now 20 years old and free of disease.

### 3.2. Histology and Immunohistochemistry

Morphologically normal primordial follicles in ovarian tissue from Patient 1, Patient 2, and Patient 3 were identified at a mean density of 55.7 (SD 14.6), 45.2 (SD 12.1), and 56.2 (SD 7.4) follicles per mm^3^, respectively.

**Figure 2:**
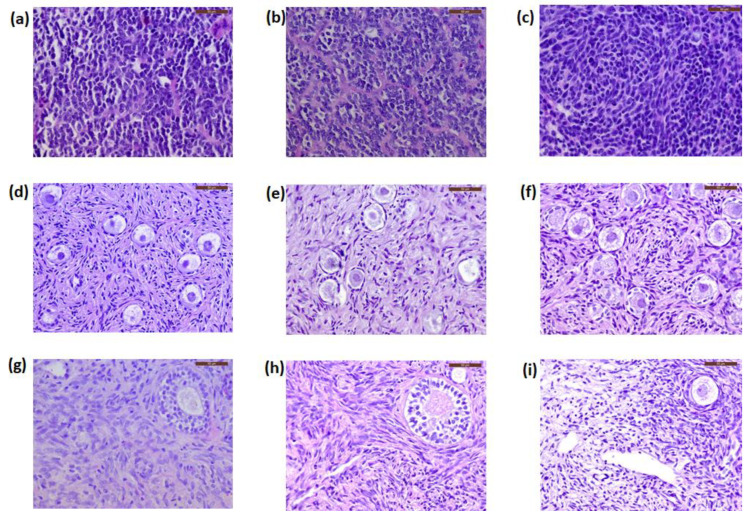
Histological sections of the primary tumors (primitive neuroectodermal tumor, PNET) and ovarian tissue from the patients. Primary tumors from Patient 1 (**a**), Patient 2 (**b**), and Patient 3 (**c**) are characterized by small round blue cells and rosette-like structures. Frozen–thawed ovarian tissue (**d**–**f**) and ovarian tissue after long-term grafting (**g**–**i**) showed no cancer cell contamination. Scale bars: 50 μm.

All sections were negative for malignant cell presence at histology (Figure 2d–f). The patients’ primary tumors were used as positive controls. These PNET cells, characterized by small round blue cells and rosette-like structures, are illustrated in Figure 2a–c.

**Figure 3:**
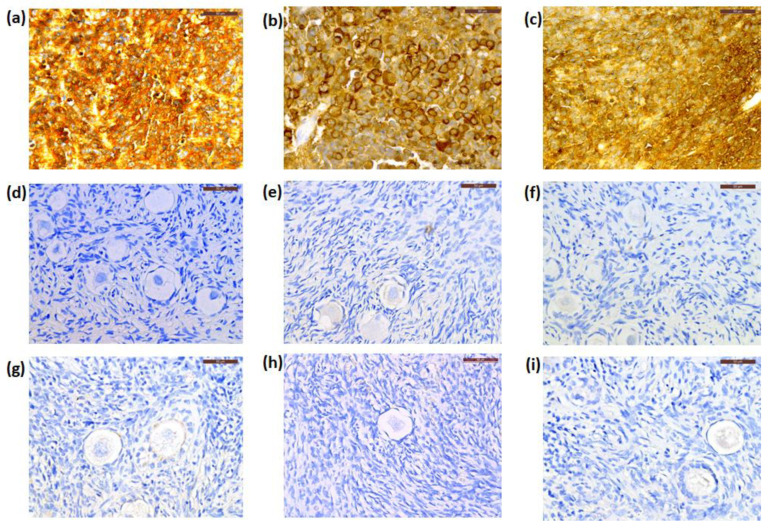
Representative photos of immunostaining for neuron-specific enolase (NSE) in three patients. The primary tumor (PNET) from Patient 1 (**a**), Patient 2 (**b**), and Patient 3 (**c**) showed NSE positive expression, while staining in frozen–thawed (**d**–**f**) and xenografted ovarian tissue (**g**–**i**) from Patients 1, 2, and 3 were respectively negative. Scale bars: 50 μm. PNET, primitive neuroectodermal tumor.

**Figure 4:**
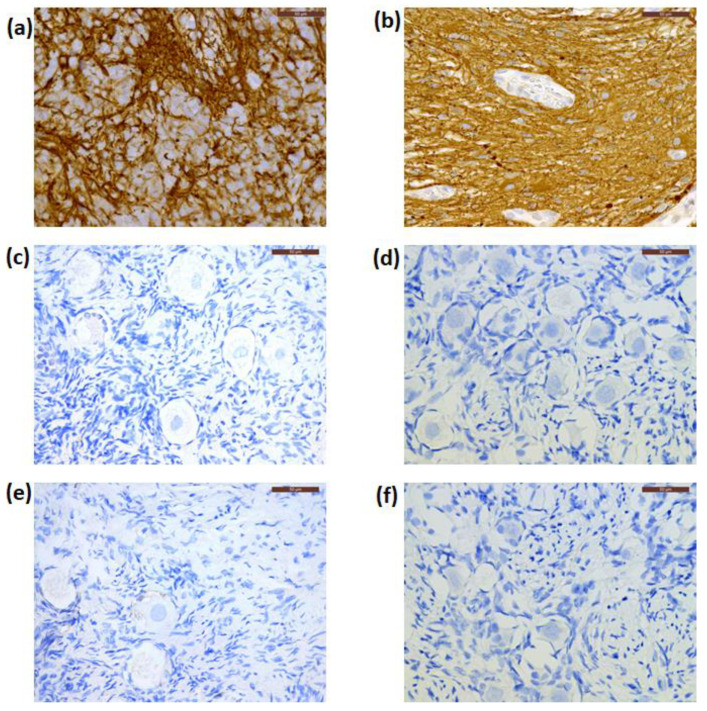
Immunostaining for glial fibrillary acidic protein (GFAP) in the primary tumor, and cryopreserved and xenografted ovarian tissue from Patient 2 (**a**,**c**,**e**) and Patient 3 (**b**,**d**,**f**), respectively. These ovarian tissue fragments showed negative expression for GFAP. Scale bars: 50 μm.

By IHC, cryopreserved ovarian cortical tissues from all patients were negative for NSE expression (Figure 3d–f), while this marker was extensively expressed in their primary CNS tumors (Figure 3a–c) and Patient 1′s recurrent tumor. The primary tumor from Patient 1 was negative for GFAP immunostaining, while those from Patient 2 and Patient 3 were positive for GFAP expression (Figure 4a,b). Cryopreserved ovarian tissue from Patient 2 and Patient 3 also showed no GFAP immunoexpression (Figure 4c,d).

### 3.3. Droplet Digital PCR

The LOB of ENO2 in human ovarian tissue was defined by measuring the level of gene expression of 60 replicates of pooled normal ovarian samples from 10 healthy women, considered as negative controls, and yielded 28.5 copies/µL. As there was no significant difference between ENO2 concentrations in negative controls and the patients’ primary tumors (positive controls, Supplementary Materials Figure S2), the ENO2 gene could not be used to determine the presence of PNET cell contamination of their ovarian tissue. Indeed, positive signals for ENO2 were also obtained in normal ovarian cortex from 10 healthy women.

**Figure 5:**
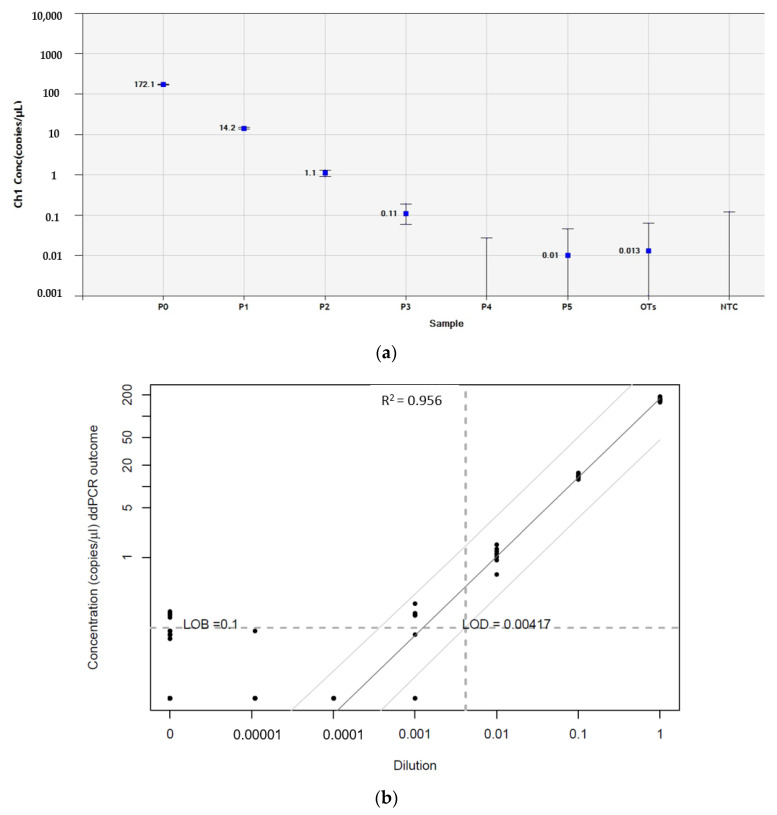
Determination of the limit of blank (LOB) and limit of detection (LOD) of GFAP in human ovarian tissue from patients with PNET by reverse-transcription droplet digital polymerase chain reaction (RT-ddPCR). (**a**) Standard dilutions between primary tumors from Patient 2 and Patient 3 and pooled samples of 10 normal ovarian tissue pieces. The *x* and *y* axes represent concentrations and dilutions, respectively. (**b**) The LOB of GFAP in normal ovarian tissue was 0.1 copies/µL. LOD values for GFAP determined by serial dilutions between PNET samples (Patient 2 and Patient 3) and pooled samples of normal ovarian tissue were low, at 0.15 copies/µL with high linearity (R2 > 0.9). GFAP, glial fibrillary acidic protein; OTs, pooled samples of normal ovarian tissue from 10 healthy women; NTC, no-template control; PNET, primitive neuroectodermal tumor. Sample P1 = 100% PNET; P1 = 10% PNET + 90% OTs; P2 = 1% PNET + 99% OTs; P3 = 0.1% PNET + 99.9% OTs; P4 = 0.01% PNET + 99.99% OTs; and P5 = 0.001% PNET + 99.999% OTs.

**Figure 6:**
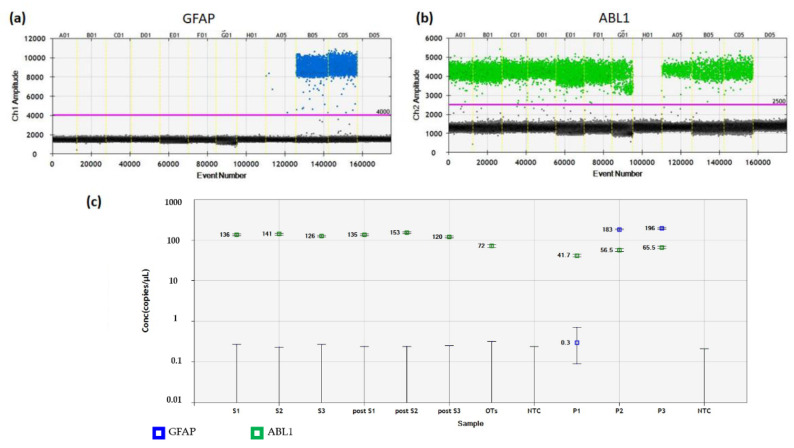
Detection of GFAP gene amplification in cryopreserved and xenografted ovarian tissue from three patients. (**a**) FAM fluorescent signals of GFAP transcripts in each droplet were plotted against the cumulative droplet count. The blue dots illustrate individual droplets containing at least one transcript copy. The black and gray dots represent negative droplets (background). No positive droplets were found in any no-template control wells (H01 and D05). (**b**) Plotting of VIC fluorescent dye for ABL1 in each well is shown. Patient samples, positive controls (PNET), and negative controls (normal ovarian tissue) contained green droplets, while no-template controls (well H01 and D05) showed no green signals. (**c**) Concentrations of samples were calculated, with blue squares representing GFAP transcripts, and green squares ABL1 amplifications. No cancer cells were detected in any ovarian tissue samples. GFAP, glial fibrillary acidic protein; PNET, primitive neuroectodermal tumor. S1–3, cryopreserved samples from Patients 1–3; post-S1–3, xenografted samples from Patients 1–3; P1–3, primary tumor from Patients 1–3 (positive controls); OTs, pooled samples of normal ovarian tissue from 10 healthy women (negative controls); NTC, no-template control.

By contrast, the LOB of GFAP in human ovarian tissue was low, yielding 0.1 copies/µL. GFAP LOD values for detecting the presence of PNET cells in ovarian tissue from Patients 2 and 3 were 0.004 and more accurately resulted in 0.14 GFAP copies/µL (Figure 5). Levels of GFAP gene expression were quantified absolutely by RT-ddPCR in cryopreserved ovarian tissue from Patient 2 and Patient 3, as illustrated in Figure 6. No GFAP transcripts were detected in these samples, while concentrations of positive controls were 183 (Patient 2) and 196 (Patient 3) copies/µL.

### 3.4. Next-Generation Sequencing

The purpose of NGS is to identify any mutations specific to the primary cancer, so this was investigated in Patient 1. If available, these mutations may be subsequently used as markers to detect possible cancer-cell spread to ovarian tissue. However, no mutations of genes of interest in the glioma panel were detected by NGS in Patient 1′s primary tumor, so NGS analysis was not conducted on her ovarian samples.

### 3.5. Xenotransplantation

After five months of xenografting and frequent follow-up, there was no sign of disease in any of the SCID mice used for this study. No suspicious masses were encountered in transplanted sites, upon macroscopic evaluation. All the grafted tissue fragments had decreased in size. Human ovarian fragments from PNET patients were de-grafted from the mice and investigated.

Ovarian follicles were observed at different developmental stages. Follicle density in ovarian tissue from Patient 1, Patient 2, and Patient 3, after grafting, was 21.4 (SD 9.9), 16.4 (SD 6.5), and 22.3 (SD 7.7) primordial follicles/mm^3^, respectively. None of the serial sections from the patients’ ovarian tissue showed any evidence of cancer cells by histological analysis (Figure 2g–i). Similarly, none of the samples expressed NSE immunostaining (Figure 3g–i).

In xenografted ovarian tissue from Patient 2 and Patient 3, GFAP expression was also negative at IHC analysis (Figure 4e,f). In addition, these samples showed no GFAP gene amplification by ddPCR (Figure 6). The absence of GFAP-positive droplets in any of the no-template controls and the stable appearance of ABL1 housekeeping gene transcripts in this duplex ddPCR run highlighted the reliability of the test. Patient information is detailed in Table 1.

In summary, no malignancy reseeding was detected in the frozen–thawed ovarian tissue of any of our subjects by histology or IHC before or after long-term xenotransplantation. Analysis by RT-ddPCR also identified no GFAP transcripts in the cryopreserved and xenotransplanted ovarian tissue from Patient 2 and Patient 3.

## 4. Discussion

Of the three subjects involved in this study, Patient 1 underwent reimplantation of her ovarian tissue seven years after cryopreservation. Following transplantation, spontaneous menstruation resumed after four months, which is consistent with the literature. Indeed, recovery of ovarian function after grafting generally takes four to five months and is achieved in more than 95% of cases [1,2,3,5]. Patient 1 gave birth to three healthy babies by natural conception between 2009 and 2012, making her one out of just three patients to obtain three successive pregnancies and live births worldwide, following one ovarian tissue transplantation attempt [20,21]. Due to recurrence of her original cancer, she died. Questions were raised as to whether this recurrence was the inevitable evolution of the primary cancer or linked to the reimplantation of ovarian tissue. Thorough investigations and evaluations were therefore carried out to exclude the presence of malignant cells in the frozen–thawed ovarian tissue of this patient with CNS-PNET.

CNS-PNETs are rare, highly aggressive neoplasms, representing only 2–3% of all childhood brain tumors [22]. Based on the 2007 World Health Organization (WHO) classification, PNETs are a group of embryonal tumors consisting of poorly differentiated or undifferentiated neuroepithelial cells, which can proliferate into neuronal cells, astrocytes, ependymal cells, melanoma cells, and myocytes [23]. This makes them difficult to diagnose by routine histopathology [24]. In the most recent WHO classification of CNS tumors (2016), the term primitive neuroectodermal tumor or PNET was actually removed from the diagnostic glossary [25]. However, the cases reported here occurred prior to 2016, and the pathological diagnoses were confirmed on the basis of previous criteria, which is why the term PNET is still used here to describe this disease.

Despite improvements in treatment modalities, the prognosis of supratentorial PNET has been historically poor. Recurrence of malignancy is observed in 37–56% of patients and is considered to be the main cause of patient mortality [26,27,28,29,30]. The interval to recurrence usually ranges from 3 to 94 months [28,29,31]. A study by Perreault et al., in 2013, revealed that 50% of relapsed patients did not respond to treatments and often died 6 to 28 months after diagnostic confirmation of their recurrence [28].

It is known that a certain proportion of relapsing patients will eventually develop diffuse leptomeningeal dissemination, which may be related to genetic mutations [28,29]. Patient 1 in our study suffered a local relapse 12 years after her last chemotherapy session and six years after ovarian tissue reimplantation. She was diagnosed with intracranial recurrence on the frontal lobe and underwent multi-therapeutic management. However, four months after completion of treatments, the disease evolved, and she developed leptomeningeal diffusion and medullary compression, leading to death. Patient 2 died in similar circumstances, with the spread of meningeal nodules.

Extracranial metastases of CNS-PNET are infrequently reported in the literature. Most commonly mentioned sites of metastasis are regional lymph nodes, lungs, and vertebral bones [32,33,34]. Our Patient 1 also suffered pulmonary metastasis one year after complete resection of her primary tumor. Metastases regularly occur in patients undergoing cranial surgery, suggesting that local mechanical barriers may play a crucial role in disseminating cancer cells outside the CNS [33,35]. A likely explanation is that craniotomy may rupture vascular channels, resulting in the spread of malignant cells through blood and lymph vessels to extracranial sites. Peritoneal seeding of CNS cancers has also been observed, especially in patients with VP shunt insertion [15], because this shunt acts as an initial pathway for cancer cells in cerebrospinal fluid to spread to the abdominal cavity. Patient 3 in our study, a prospective candidate for ovarian tissue transplantation in the future, received a VP shunt before ovarian tissue collection and cryopreservation. Testing for MDD in her ovarian tissue was therefore vital. Our investigations showed that her cryopreserved and xenografted ovarian tissue was not contaminated by malignant cells, as confirmed by histology, IHC, and ddPCR. Indeed, metastasis of CNS-PNET to the ovaries has not yet been reported in the literature, but several cases of peripheral PNET originating in the ovaries have been published (Table 2). It should be borne in mind, however, that cancers in these cases primarily stemmed from the ovaries, without any tumors in the CNS itself, which differs from our current cases.

Recurrence of the original cancer after transplanting ovarian tissue has been recorded in several studies. A review of worldwide data on ovarian tissue transplantation in 2018 reported that 9 out of 230 women with malignant disease experienced a relapse after ovarian tissue grafting, but none of the cases was thought to be related to the reimplantation procedure [14].

The current study has a small sample size, so further well-designed and conducted research is needed to confirm the quality of our data. While there is currently no evidence to suggest that ovarian tissue transplantation causes reseeding of the primary cancer [14,44], the risk of ovarian metastasis cannot be completely ruled out for any type of tumor, because of the lack of highly specific detection methods at the time of grafting and the possibility of sampling error due to bias [45].

## 5. Conclusions

In the present study, it is important to emphasize that Patient 1′s transplanted ovarian tissue was confirmed to be without any detectable malignancy, by histological analysis, at the time of ovarian tissue cryopreservation and transplantation. Her remaining frozen tissue showed no contamination by cancer cells in histological sections, either after thawing or long-term xenotransplantation. This ovarian tissue was also negative for NSE expression, even though this marker was strongly expressed in the patient’s primary tumor and recurrent tumor. Furthermore, no malignancy was detected in cryopreserved ovarian tissue from the other two patients by histology, IHC for NSE and GFAP, RT-ddPCR for detection of GFAP gene transcripts, or xenotransplantation to SCID mice. Indeed, we did not find any relationship between the primary cancer relapse and reimplantation of the patients’ cryopreserved ovarian tissue.

To conclude, no malignancy was detected in any ovarian tissue samples from our patients with CNS-PNET, but relapse and disease progression are more likely to occur with CNS-PNET, as it is the nature of the disease. In this study, there was no evidence to associate the recurrence of primary cancer with reimplantation of ovarian tissue.

## Figures and Tables

**Table 1:** Details of CNS-PNET patients who underwent ovarian tissue cryopreservation.

Patient n°	Age * (years)	Extraneural Metastasis	Relapse	Alive or Deceased	Cancer Cells in Histology	Immunohistochemistry			Concentration of GFAP Transcripts by RT-ddPCR (Copies/µL)	
						Patient CNS Tumors	Cryopreserved OT	Xenografted OT	Cryopreserved OT	Xenografted OT
1	17	Yes, lungs	Yes	Deceased	No	GFAP (−), NSE (+)	NSE negative	NSE negative	0	0
2	4	No	No	Deceased	No	GFAP (+), NSE (+)	Both negative	Both negative	0	0
3	9	No	No	Alive	No	GFAP (+), NSE (+)	Both negative	Both negative	0	0

(*) Age at ovarian tissue collection; CNS, central nervous system; GFAP, glial fibrillary acidic protein; NSE, neuron-specific enolase; OT, ovarian tissue; PNET, primitive neuroectodermal tumor; RT-ddPCR, reverse-transcription droplet digital polymerase chain reaction.

**Table 2:** Case reports on peripheral primitive neuroectodermal tumors of the ovary.

Authors	Patient Age (Years)	FIGO Stage	Treatment	Complications	Follow-Up
Kawauchi et al. (1998) [36]	29	II	TAH + BSO + omentectomy + PALA + chemotherapy	NA	11 months DOD
Chow et al. (2004) [37]	13	IV	Debulking + chemotherapy 2nd debulking + chemotherapy + radiotherapy	NA	17 months DOD
Demirtas et al. (2004) [38]	25	IC	LSO + omentectomy + PLA + chemotherapy 2nd look laparotomy	Pelvic abscess after 2nd look laparotomy	2 years 2 births NED
Kim et al. (2004) [39]	18	IIIC	RSO + omentectomy + PLA + PALA + chemotherapy + radiotherapy	Bowel obstruction	10 months DOD
Ateser et al. (2007) [40]	28	IV	TAH + LSO + omentectomy + chemotherapy + radiotherapy	Neutropenia	13 months DOD
Anfinan et al. (2008) [41]	31	IIIC	TAH + BSO + omentectomy + chemotherapy	NA	15 months DOD
Ostwal et al. (2012) [42]	28	NA	LSO + chemotherapy + radical excision upon recurrence	NA	18 months DOD
Huang et al. (2013) [43]	28	IA	LSO + omentectomy + PLA + chemotherapy	NA	28 months NED

BSO, bilateral salpingo-oophorectomy; DOD, died of disease; FIGO, Fédération Internationale de Gynécologie et d’Obstétrique; LSO, left salpingo-oophorectomy; NA, not available; NED, no evidence of disease; PALA, para-aortic lymphadenectomy; PLA, pelvic lymphadenectomy; RSO, right salpingo-oophorectomy; TAH, total abdominal hysterectomy.

## Supplementary Materials

The following are available online at https://www.mdpi.com/2077-0383/9/12/4101/s1. Supplementary Table S1: Primer and probe sequences. Supplementary Figure S1: Representative photo of Patient 1′s right ovary, observed at the time of laparoscopic ovarian tissue transplantation. Supplementary Figure S2: Comparison of ENO2 gene expression between blank samples and patient primary tumors.
