# Supplementary material for: Is Ovarian Tissue Transplantation Safe in Patients with Central Nervous System Primitive Neuroectodermal Tumors?
Source: J Clin Med. 2020 Dec 18;9(12):4101. doi: 10.3390/jcm9124101 (PMC7766375; doi:10.3390/jcm9124101)
Supplement: Supplementary file 1 [file jcm-09-04101-s001.pdf]

Supplementary Table S1. Primer and probesequences.

Gene	Probe	Forward primer	Reverse primer
ENO2	5′-FAM-ATCCTGCCTGTGCCGGCCTTC-3′	5′-GGGAACTGCCCCTGTATCG-3′	5′-CATGAGAGCCACCATTGATCA-3′
GFAP	5′-FAM-AGATCGCCACCTACAGGA -3′	5′-GGCCCGCCACTTGCA-3′	5′-GGGAATGGTGATCCGGTTCT-3′
ABL1	5′-VIC-CAACACCCTGGCCGAGTTGGTTCAT-3′	5′-CAACACTGCTTCTGATGGCAA-3′	5′-CGGCCACCGTTGAATGAT-3′
B2M	5′-FAM-ACATGTCTCGATCCCAC-3′	5′-TGACTTTGTCACAGCCCAAGATA-3′	5′-CGGCATCTTCAAACCTCCA-3′

ABL1, Abelson murine leukemia viral oncogene homolog 1; B2M, beta 2 microglobulin; ENO2, enolase 2; GFAP, glial fibrillary acidic protein.

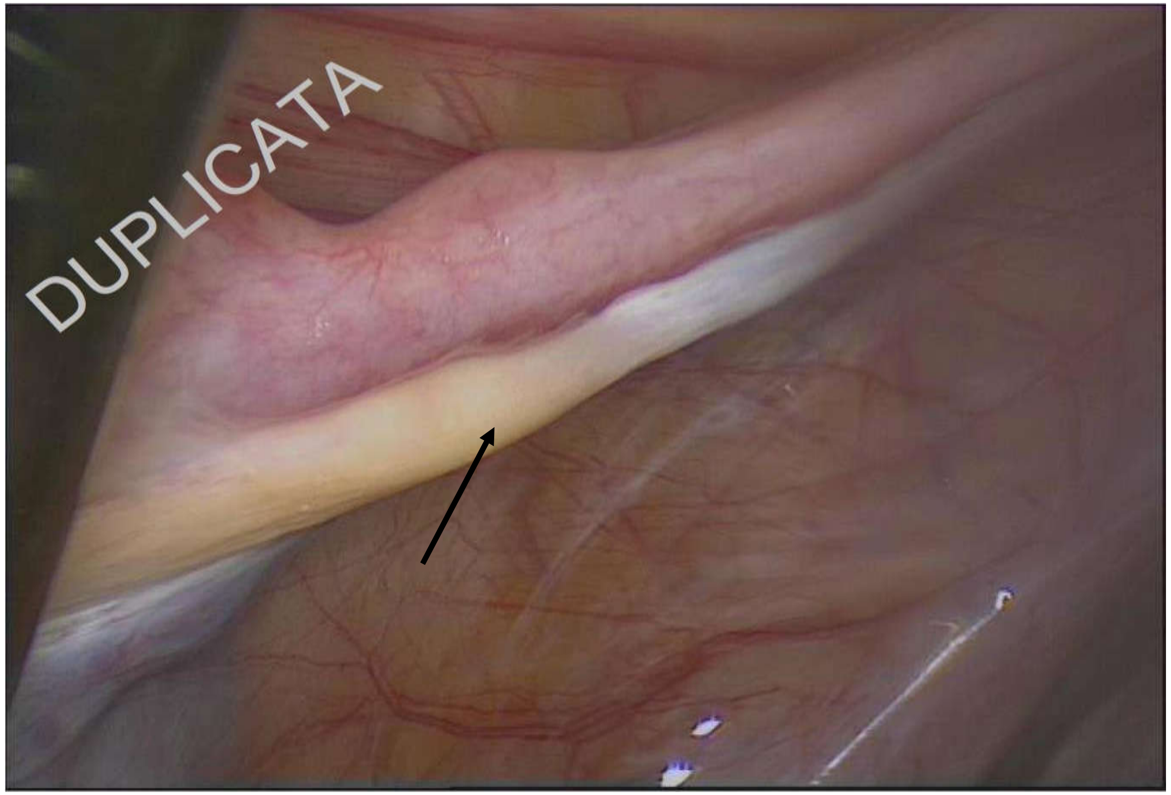

SupplementaryFigureS1. Representative photo of Patient 1's right ovary,observed at the time of laparoscopic ovarian tissue transplantation. This ovary (black arrow) was atrophic.

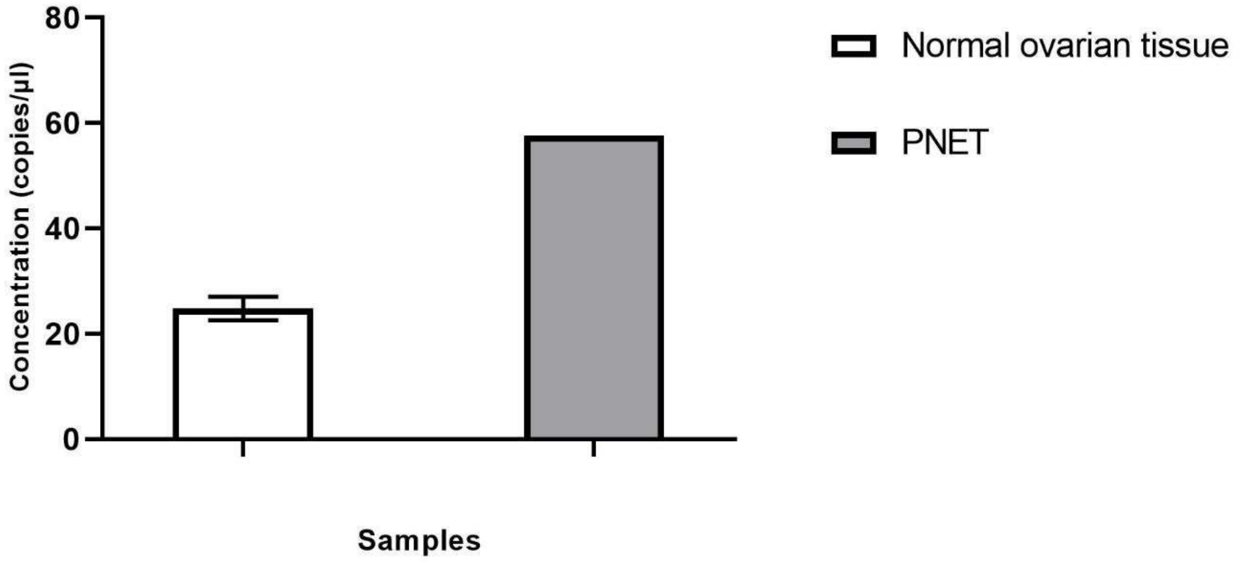

Supplementary Figure S2. Comparison of ENO2 gene expression between blank samples and patients' primary tumors. It is noticed that normal ovarian tissue (blank samples) produced ENO2 genes at moderate levels, making it insignificantly statistically different from the positive control (patients' primary tumors). ENO2: enolase 2.
